# Effect of Germination on Seed Protein Quality and Secondary Metabolites and Potential Modulation by Pulsed Electric Field Treatment

**DOI:** 10.3390/foods13111598

**Published:** 2024-05-21

**Authors:** Norma Cecille Bagarinao, Jessie King, Sze Ying Leong, Dominic Agyei, Kevin Sutton, Indrawati Oey

**Affiliations:** 1 Department of Food Science, University of Otago, P.O. Box 56, Dunedin 9054, New Zealand; norma.bagarinao@postgrad.otago.ac.nz (N.C.B.); jessie.king@otago.ac.nz (J.K.); sze.leong@otago.ac.nz (S.Y.L.); dominic.agyei@otago.ac.nz (D.A.); 2 Riddet Institute, Private Bag 11 222, Palmerston North 4442, New Zealand; kevin.sutton@plantandfood.co.nz; 3 The New Zealand Institute for Plant & Food Research Limited, Private Bag 4704, Christchurch Mail Centre, Christchurch 8140, New Zealand

**Keywords:** seeds, germination, water uptake, plant protein, secondary metabolites, antioxidant, sprouted foods

## Abstract

Plant-based foods are being increasingly favored to feed the ever-growing population, but these need to exhibit improved nutritional value in terms of protein quality and digestibility to be considered a useful alternative to animal-based foods. Germination is essential for plant growth and represents a viable method through which the protein quality of plants can be further improved. However, it will be a challenge to maintain efficient rates of germination in a changing climate when seeds are sown. In the context of the indoor germination of seeds for food, consumption, or processing purposes, a more efficient and sustainable process is desired. Therefore, novel techniques to facilitate seed germination are required. Pulsed electric fields (PEF) treatment of seeds results in the permeabilization of the cell membrane, allowing water to be taken up more quickly and triggering biochemical changes to the macromolecules in the seed during germination. Therefore, PEF could be a chemical-free approach to induce a stress response in seeds, leading to the production of secondary metabolites known to exert beneficial effects on human health. However, this application of PEF, though promising, requires further research to optimize its impact on the protein and bioactive compounds in germinating seeds.

## 1. Introduction

With the global population expected to exceed nine billion people within the next 30 years, the Food and Agriculture Organization (FAO) [1] estimates that a 50% increase from 2012’s food production will be needed to provide adequate food for all. This rapid growth in the population, along with increasing consumption trends, will increase emissions of climate-changing greenhouse gases. The prevailing opinion posits that the production of crops for food is more environmentally sustainable than that of animals [2]. These comparisons are commonly based on land and water use, energy conversion, greenhouse gas emissions, and mean protein intakes [3,4]. Contrary to this, others have argued that the efficiency and sustainability of plant versus animal agriculture should include considerations of nutritional quality and composition [5]. Animals are a major source of dietary proteins, providing the body with a range of indispensable amino acids that are vital for metabolism, growth, and development [5]. It is widely viewed that plant-derived proteins are nutritionally insufficient when compared to animal-based proteins, as they are low in certain indispensable amino acids and have lower digestibility for humans and monogastric animals. However, plant proteins can be rich sources of fiber and bioactive compounds, which are known to be beneficial for human health [6,7]. Owing to the lower production cost of plants and the easy access to them in many parts of the world, plant-based proteins present a promising solution to maintain food production sustainability and security. However, the use of green technologies to improve their nutritional quality is essential for them to be considered suitable alternatives to animal-derived proteins.

Germination is an essential stage of plant growth, in which seeds exit dormancy to produce seedlings. Furthermore, the metabolic adaptations occurring in seeds during this time, resulting in foods with improved protein quality and other nutritional parameters, sets up the potential for germinated seeds to be used as functional foods. For example, the consumption of pre-germinated cooked brown rice (100 g/day as a replacement for white rice) by 85 patients with type two diabetes was found to reduce fasting blood glucose levels, glycated hemoglobin, and triglyceride levels, while increasing high-density lipoprotein levels and improving the fatty acid profile towards a higher n3:n6 ratio [8]. Similarly, Bui et al. [9] reported that 60 women (45–65 years) with impaired glucose tolerance consuming pre-germinated cooked brown rice instead of white rice for four months showed improved blood glucose levels, body mass index, and blood pressure. Additionally, germinated seeds can be eaten raw as sprouts or milled into flours that can be incorporated into the production of other food products (e.g., bread, cereals, milk alternatives, and yoghurt) to enhance their nutritional value [10].

A major concern for future food security is the ability to efficiently produce foods in changing environmental conditions [11]. Increased temperatures, drought, flooding, and contamination present serious risks to plant germination, all of which are expected to become more prevalent. A critical moisture content must be attained for germination to proceed [12]. This can be achieved by soaking the seeds in water as a preconditioning step to ensure rapid growth and germination [13]. However, soaking may inhibit, delay, or hasten germination, depending on the duration, sensitivity, and moisture requirement of the seed [12,13,14]. Thus, there is an urgent need for technologies that can impact seeds’ water uptake to improve the success and efficiency of germination. Various novel technologies have been studied for their potential in food processing and breaking seed dormancy to improve seed germination. For example, pulsed electric fields (PEF), microwave radiation, magnetic fields, ultrasound, non-thermal plasma, UV-light A and C, ozone, plasma-activated water, and electrolyzed oxidizing water have all shown positive effects on seed germination [15]. It would be advantageous if such technologies could also increase the nutritional value of the seed by improving the protein quality and content of beneficial secondary metabolites in germinating seeds.

**Figure 1:**
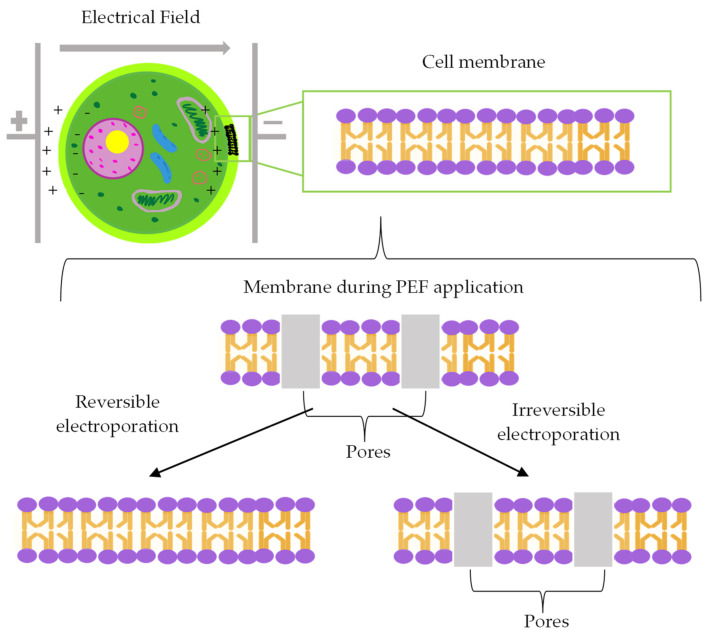
The schematic mechanism of membrane permeabilization induced by an external electric field showing reversible and irreversible electroporation.

A potential technology that could meet the aforementioned requirements is PEF, a non-thermal technology that involves the application of short, high-voltage pulses to biological materials of interest. The treated sample is placed between two electrodes over which an electric field is applied for very short durations of microseconds to milliseconds [16]. Typically, the process induces cell membrane permeabilization, also known as electroporation [17]. In this process, a transmembrane potential is induced in the cytoplasmic membrane, which initiates pore formation. Depending on the electric field intensity, the permeabilization can be either reversible or irreversible (Figure 1), allowing the technology to be used for a wide range of purposes [15,18]. This technology has been thoroughly investigated for its use in food production and processing [15]. Studies show that it uses less energy than thermal treatments, is less time consuming, preserves the quality of the product, and is highly scalable [19]. PEF has commonly been used for microbial and enzyme inactivation, cell disintegration and extraction, the improvement of mass transfer that aids in diffusion-based operations, and modifying the techno-functionality of foods [18]. Additionally, low to mild PEF treatments have been applied to stimulate stress responses that lead to the accumulation of secondary metabolites or the stimulation of growth in plants [20,21]. In the context of facilitating seed germination, the applied PEF process parameters should induce a reversible cell permeabilization that does not cause a leaching of nutrients or permanent damage and preserves cell viability [20,22]. This is crucial to enhance water uptake and stimulate the production of secondary metabolites, improving the efficiency of germination, the nutrient profile of the resulting seeds, and the subsequent foods made from them. This review will highlight how germination can be used to improve the protein quality and secondary metabolite content in germinating seeds. Furthermore, the potential uses of PEF on both dried and imbibed seeds prior to germination, as well as important considerations regarding their application, will be discussed.

## 2. Seed Germination

**Figure 2:**
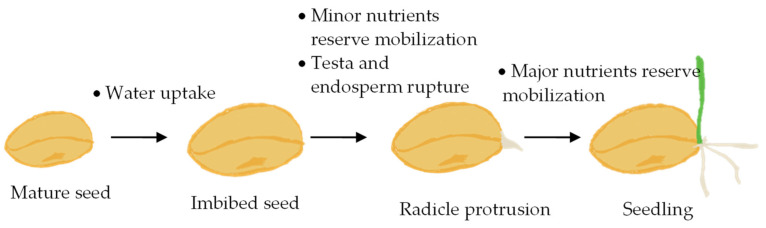
The seed germination stages.

Germination starts when a seed begins to uptake water, also known as imbibition (Figure 2). For germination to be successful, the embryo growth potential must increase and the tissues surrounding it must be weakened to allow the radicle to protrude [23]. Various hormonal and environmental factors can influence either the growth potential of the embryo or the strength of the enclosing tissues, consequently inhibiting germination. Strictly speaking, germination is complete when the radicle emerges from the structure enclosing it (Figure 2), and the processes that follow radicle emergence, including seedling growth, are referred to as post-germination processes [23]. However, most studies focusing on the use of germination to process seeds for food consumption refer to the end-product as “germinated” or “sprouted” seeds, regardless of the duration between the first radicle emergence and the harvesting point.

In most studies that utilize germination to improve the nutritional quality of edible seeds, three main steps are involved: sterilization, soaking, and sprouting. After an initial clean to remove dirt and broken seeds, sterilization follows. This step is conducted to prevent microbial growth and is usually performed prior to soaking and sprouting, during which the conditions are favorable for microbial growth. Sterilization can be achieved by soaking the seeds in solutions of formaldehyde (0.2%), sodium hypochlorite (0.07–7%), mercuric chloride (10%), ethanol (70%), or combinations of ethanol and sodium hypochlorite or ethanol and calcium chloride (3%) for 20 s to 40 min (Table S1). The seeds are then thoroughly rinsed with water to remove residual chemicals. This is followed by soaking, usually in distilled or deionized water at a seed to water ratio of 1:4–1:5 (*w*/*v*). Soaking is commonly conducted between 12 and 24 h, though some studies have reported soaking times as short as 4–5 h, at temperatures from 22–30 °C. Other considerations in the soaking set-up include a predetermined moisture content endpoint, a wet and dry phase cycle [24,25], aeration [26], and the changing of the soaking solution [27]. Finally, the seeds are sprouted, often referred to as the germination step. Soaked seeds are drained and placed in a thin layer on trays or containers with water-saturated cloth or paper. They are then incubated in a controlled environment, generally at 20–30 °C, for anywhere between 1 and 9 days.

### Seed Soaking and Water Uptake

Water uptake signals the resumption of metabolism and the initiation of cellular events during germination and is driven by the water potential gradient, i.e., the external water potential being higher than the water potential of the seeds. The imbibition of dry seeds with a water potential of −350 to −50 MPa in pure water (which has zero water potential) allows for a very high potential gradient, thus, imbibition ensues [23]. Given that the water distribution is not uniform among seed tissues, water enters more rapidly through the most permeable region of the seeds, usually the micropylar region in cereals and other seeds, and the mobilization of seed storage reserves begins in areas with high water densities [28]. Water uptake occurs in three phases; phase I is characterized by the initial rapid water uptake, followed by a plateau (phase II), and the resumption of water uptake (phase III) accompanied by embryo growth. Water uptake by the embryo is essential for radicle emergence, as the embryo must expand and exert pressure on the surrounding tissues. As such, germination can be inhibited if the water supply to the seed is reduced or if the water potential of the imbibition solution is too low [28]. Dry seeds generally have a moisture content in the range of 5–15%, while fully imbibed seeds have a moisture content of 75–100% on a dry weight basis (5–13% and 40–50% moisture contents on a fresh weight basis, respectively) [23]. The rate of water uptake during phase I is dependent on the permeability of the seed coat or the testa, which contains lignified cells and a waxy covering, limiting the amount of the water that can reach the embryo, therefore regulating germination. Indeed, some seeds with a hard testa are resistant to imbibition unless scarred or damaged. On the other hand, damage to and the removal of the testa can cause rapid imbibition, leading to the disruption of cell walls, the blistering of the cotyledon surface, and the leakage of cellular contents into the soaking medium which consequently kills the seed [23].

The differences in water uptake between seeds means their optimal soaking duration can vary widely. For example, among 10 cultivars of lychee seeds, the soaking time required to achieve 100% germination varied from 26–54 h [29]. Broad beans that were soaked for up to 72 h exhibited faster germination times while pea, sunflower, and common bean germinated slowly and were less vigorous after 24 h of soaking. The common bean has been found to be more water-sensitive, with just 6 h of soaking negatively impacting growth [13]. This was attributed to a reduced oxygen supply to the axis due to excess water. In an anaerobic environment, ethanol is produced which may cause the self-poisoning of the seed. Furthermore, soaking has also been reported to slow down the synthesis of the RNA and protein in an *Agrostemma githago* embryo [30]. However, for other seeds, such as rice, oat, pea, and lettuce, soaking has little effect [13]. The soaking conditions (i.e., the duration, temperature, humidity, and seed-to-water ratio) required for optimal germination therefore need to be tailored to the seed in question.

## 3. Effects of Germination on Seed Nutrients

The mobilization of seed storage reserves during germination induces a number of changes to macronutrients. The activation of amylases, for example, leads to the hydrolysis of complex starch molecules in seeds, degrading them into simple sugars. A reduction in the total carbohydrates and starch with a concomitant increase in sucrose and a reduction in sugars has been reported for germinated seeds [31,32,33]. In addition, germination can change the starch components by reducing slowly digestible starch, increasing rapidly digestible starch [34], and changing the starch structure [32]. Similarly, germination changes the lipid content and fatty acid profile of the seeds, though the effects vary depending on the seed type and germination conditions. For example, both reductions and increases in lipid contents have been reported for legumes and cereal grains [31,35]. Furthermore, an increase in polyunsaturated fatty acids and a decrease in saturated fatty acids has been reported in various sprouted grains [35]. Germination can also affect seed micronutrients; increases in β-carotene, vitamin C, and vitamin B contents [31,36] and in the enhanced bioavailability of Zn, Cu, Mn, and Fe have been reported for germinated seeds [36]. For a more detailed review on the impact of germination on these nutrients, the reader is directed to a recent review by Gunathunga et al. [32]. However, in the context of sustainable food production and food security, the efficient production of quality protein presents the greatest challenge [5,37]. Thus, the potential of germination to improve the protein content, quality, and digestibility of plants must be considered.

### 3.1. Effects of Germination on Seed Proteins

Proteins are broken down and re-synthesized during germination, minorly to support the radicle’s emergence and primarily to support seedling growth. Significant changes in seed protein content owing to germination have been reported, but the extent varies (Table S2), with increases as low as 2% being reported for a hemp protein isolate [38] and as high as 94% for mung bean flour [31]. Variations in the results of different studies are influenced by differences in botanical sources (species, genotypes, cultivars), soaking and germination treatments (length, temperature, lighting, other conditions), the sample preparation prior to analysis, and the protein assay method (including the conversion factor used to covert nitrogen to protein estimates). In general, however, the protein content of germinated seeds tends to increase as the germination time is lengthened [31,38,39,40,41,42,43,44,45,46,47,48,49,50]. This trend can be observed in separate studies conducted on the same species. For example, in oats, the protein content of grains germinated for 2, 3, and 8 d increased by 1.4% [51], 10.4% [26], and 12.3% [40], respectively. Likewise, the protein content of mung bean flour in different studies was observed to increase by 12.6% after 3 d [48], 39.2% after 4 d [49], and 94.3% after 5 d [31].

However, the germination duration required to achieve an optimum protein content may vary between different seed species, varieties, and genotypes. For example, amaranth seeds germinated for 18 h accumulated 11% more proteins than ungerminated seeds. Extending the germination time to 24 h significantly decreased the protein content, though it was still 7.6% higher than the control [52]. Similarly, the protein content of finger millet var. of Tessema seeds was reported to decrease by 35% when germination was extended from two days to three days [43]. This characteristic is different from the finger millet seeds of other varieties reported in the same paper, which attained maximum protein contents after three days. In three separate studies, some genotypes of soybeans (Nova, Adasoy, Turksoy, Nazlican, A3127, ATEM-7, and A3935) that were germinated for 10 days attained a lower percentage increase in protein contents [53] than soybeans (*Glycine max* L.) germinated for three days [45,54] and *Glycine max* L. var. of Woodworth germinated for five days [44]. The mobilization of seed storage proteins during germination can influence the protein quality of the germinated seeds in many ways. Enzymes in the seeds that are activated upon imbibition break down storage proteins into amino acids that are used to synthesize more proteins and other nutrients needed for seedling growth, thus increasing the protein contents of germinated seeds. Additionally, the de novo synthesis of enzymes and other proteins may also contribute to an increase in protein content. However, these changes were reported to occur in different stages of germination or post-germination [55,56,57] which may explain the difference in the protein contents of seeds germinated at different durations. A decrease in the protein content may be attributed to the degradation of proteins to be utilized for other purposes, such as carbon skeletons for respiratory oxidation or conversion into other metabolites [57].

Temperature also affects protein accumulation during germination. For example, a study on the optimal conditions for oat germination reported optimum steeping and germination temperatures of 18 °C and 20 °C, respectively. Lower and higher temperatures decreased the total soluble protein content of the seeds germinated for two days [58]. Few studies, however, have investigated the effect of temperature on germination with respect to changes in protein quantity and quality. Studies on optimizing germination temperature are mostly aimed at improving the germination rate and capacity, the seed growth, and establishment. Seeds can germinate in a wide range of temperatures [59], but an optimum temperature can be identified based on the ideal germination rate and capacity. For example, legumes were reported to attain a germination capacity of greater than 70% after 14 days of germination under temperatures ranging from 15–30 °C, depending on the species [59]. This may not be ideal for industry, as a 14-day germination time would entail a long production cycle, ultimately increasing production costs. The mechanism by which temperature influences protein accumulation has not been elucidated in studies relating to germinating seeds for food, consumption, and processing. However, temperature can influence the activity of different enzymes involved in germination and post-germination processes, which could impact the mobilization of storage reserves, including proteins, in seeds. It can also affect water uptake and, consequently, the rate of germination [60].

Germination duration has an important influence on the protein content of products from sprouted seeds, but the optimal germination time varies with different species and is confounded by many factors ranging from inherent differences in the genotypes or cultivars, soaking conditions, and other germination conditions, including temperature. It should also be noted that an increase in non-protein nitrogen has also been reported for germinated seeds [40,61], which may overestimate the protein content reported in the literature. Furthermore, extended germination times increase the risk of mold growth. Even with sterilization in a 0.2% formaldehyde solution, mold growth was observed in 5–10% of oat grains [40] and 25–50% of sorghum (dependent on variety) after six days [41]. Fungal growths were observed in amaranth and mung bean seeds that were surface sterilized after two and five days of germination, respectively. Naturally, the germination capacity increases as germination time is increased, and this could lead to an increased protein content. However, there may be a point at which the protein content begins to decline and spoilage may occur. Thus, a balance must be reached when selecting appropriate germination times, and these will likely vary depending on the seed in question.

### 3.2. Changes in the Protein Composition of Germinated Seeds

Although changes in crude protein content can be variable, changes in protein composition are commonly observed in germinated seeds (Table S2). For example, germinated oat seeds exhibited an increase in soluble nitrogen and free amino acids, but crude protein remained the same [24]. The same trend has been reported for buckwheat seeds, although there was no change in soluble nitrogen content. Similarly, free amino acids and amino nitrogen were reported to increase in germinated soybeans without any increase in the protein quantity [62]. Likewise, an increase in free amino acids was reported for germinated sorghum despite a significant decrease in the total protein content [63].

These changes in the protein composition depend on the type of protein in the seeds. In general, germinated seeds display protein profiles with increased low-molecular-weight (LMW) peptides and decreased high-molecular-weight (HMW) peptides. For example, germinated oats exhibited an increase in albumin proteins and a decrease in globulin and prolamin proteins [24,40,64]. Similarly, an increase in albumin and a decrease in kafirin (prolamin) and glutelin was reported for germinated sorghum [41]. Germinated triticale also showed a decrease in prolamin and glutelin [42]. This behavior is attributed to the mobilization of proteins during germination that breaks down the storage proteins in the seeds. For instance, HMW peptides ranging from 25–100 kDa in chickpeas and LMW peptides of 11–19 kDa were reported to decrease and increase, respectively, after germination [65]. Similarly, electrophoretograms of lentil proteins displayed diminishing peptide bands of 33–80 kDa and increasing peptide bands of 19 and 14 kDa in lentils as germination progressed. The largest protein subunits were broken down primarily at the start of germination, and this was followed by the appearance of smaller subunits as germination lengthened. This shift towards more LMW peptides could lead to enhanced peptide absorption in the gastrointestinal tract [66], allowing more bioactive peptides to exert their physiological effects.

#### Changes in the Amino Acid Composition of Germinated Seeds

In addition to the altered protein composition, studies have reported a positive effect of germination on the amino acid contents of seeds (Table S2). These studies have shown that an increase in the protein content was accompanied by an increase in the total amino acids (TAAs) [40,42,44,52,67,68], which is associated with changes in the protein fractions. For example, an increase in the lysine-rich albumin fraction in oat seeds was accompanied by a rise in the respective amino acid. Similarly, a decrease in the globulin fractions rich in phenylalanine and tyrosine correlated to a decrease in these amino acids [40,41]. This is an important consideration for the nutritional value of germinated foods, as the amino acid profile may be more important than just quantifying the protein content. For example, Li and Xu [26] found that the crude protein in oat seeds increased as germination progressed, reaching a maximum after 72 h. On the other hand, the TAA content was at a maximum after 48 h and decreased when germination continued for 60 and 72 h [26]. A similar study on oats reported a decrease in TAAs after 24 h of germination which increased thereafter, while the protein content increased progressively from the start of imbibition [69]. Another study on oats reported a significant increase in protein content, but the TAA content decreased by 15.35% with no significant change in the total essential amino acids and a substantial decrease of 17% for the total non-essential amino acids [64]. An increase in protein content accompanied by a decrease in TAAs may be partly explained by an increase in the amount of non-protein nitrogen, as reported by other authors [42,61].

Notably, germination may also improve the essential amino acid profile. For example, oats, which are often deficient in lysine, were found to exhibit a 4.5-fold increase in lysine when germinated [58]. Interestingly, the amount of histidine, leucine, isoleucine, lysine, and valine in germinated oat seeds was higher compared to other germinated seeds (mung bean, pigeon pea, and soybean), despite all of these initially containing higher contents of the respective amino acid compared to oat seeds. Germinated wheat also exhibited an increase in lysine and isoleucine along with other essential amino acids [70]. All reported essential amino acids and non-essential amino acids also improved in the germinated pigeon pea [67]. While some amino acids reportedly decreased for the germinated amaranth [52], mung bean [31], and soybean [44], most of the essential amino acids increased, leading to an overall increase in the TAAs. Considering this, it is important to determine the optimal germination length to maximize the content of amino acids, particularly essential amino acids.

### 3.3. The Effect of Germination on the Protein Digestibility of Seeds

In addition to changes in the protein and amino acid content of seeds, germination may also be useful in enhancing protein digestibility, allowing more amino acids to be absorbed in the human body upon consumption and digestion. This is particularly important to justify the nutritional value of plant-based proteins compared to animal proteins. The increase in the protein digestibility of germinated seeds is thought to occur via four main mechanisms: (1) the hydrolysis of proteins into smaller peptides and amino acids that are more readily accessible to human digestive enzymes, (2) the enhancement of protein solubility, (3) a reduction in the amount and activity of antinutrients, and (4) the weakening of the bonds between starches and proteins, ultimately making proteins more accessible to digestive proteases [71,72].

The effect of germination on protein digestibility has generally been found to be beneficial, with a few exceptions. The extent of digestibility enhancement differs across plant sources and can be as low as 7% and as high as 75%. Chickpeas [65], amaranth [73], faba beans [71,74], millets [27], soybeans [62], sesame [75], and yellow peas [74] showed less than or equal to a 10% increase in protein digestibility after germination. On the other hand, protein digestibility increases from 14–50% have been reported for black soybeans [76], pigeon pea [67], peas [61], oats [26], the common bean [71], and sorghum [63]. The effect on digestibility differs among plant species and varieties. For example, six varieties of millet exhibited a 7–17% improvement in in vitro protein digestibility (IVPD) [27]. Similarly, several studies on soybeans reported a statistically significant enhancement between 0.43–33% [62,76,77,78].

The germination conditions, such as the time and temperature, differ among studies, contributing to the variations. For example, sesame seeds germinated for 96 h exhibited no significant changes in IVPD. However, when the germination time was reduced to 48 h, the IVPD increased significantly by 10% [75]. The increase in IVPD was attributed to changes in the amount of free sulfhydryl groups, which may alter the protein’s secondary and tertiary structure, ultimately influencing its susceptibility to digestive enzymes [75]. On the other hand, the effect of germination duration on protein digestibility may be related to the germination phase at which these changes to protein structures and compositions occur. As mentioned above, protein mobilization can occur at different stages of germination and post-germination, and this influences the protein quality of the germinated seed. Similarly, soy protein isolates from germinated soybeans with a hypocotyl length of 5 cm exhibited a 43% decrease in hydrolysis compared to those with a hypocotyl length of 3 cm. The decrease in protein hydrolysis was attributed to the resynthesis of antinutritional components of the seed as germination progressed [77], highlighting that although protein digestibility may reach a peak at a given germination time, this needs to be balanced with the contents of other compounds that can also influence the seed’s nutritional quality. For example, sprouted oat seeds exhibited a maximum IVPD after 72 h of germination, but the essential amino acid index was significantly higher at 48 h of germination (i.e., an increase of 35% compared to 23%). Moreover, the difference between the IVPD of 48 and 72 h-germinated oat seeds was only 1% [26]. Thus, in such a scenario, it would be logical to choose the 48 h germination time for its improved protein quality and reduced processing cost.

#### The Effects of Germination on the Antinutrient Contents of Seeds

Antinutritional factors are seed components that can reduce the digestibility and nutritional values of plant-based proteins. Examples of these compounds are protease inhibitors (trypsin and chymotrypsin inhibitors), phytic acid, tannins, and oligosaccharides (stachyose, raffinose, and verbascose) [79]. In general, germination leads to a substantial reduction in antinutritional factors, such as phytic acid, tannins, trypsin and chymotrypsin inhibitors, oxalates, and oligosaccharides [73]. Up to an 84% reduction in phytic acid [49] and a reduction in tannins to being below detection limits [31] were reported in germinated mung beans. Similarly, trypsin and chymotrypsin inhibitor activity have been reported to decrease by as much as 61% and 16.53% in pigeon peas and soybeans, respectively [62,67].

Germination can also be an effective method of reducing the contents of heat-stable antinutrients, such as phytic acid and tannins. For example, boiled germinated legume (lupin, soybean, and black bean) seeds exhibited no further decrease in phytic acid compared to non-heated germinated seeds, but the activity of trypsin inhibitor, a heat-sensitive antinutrient, was reduced to a level below the limit of detection [80]. Comparing the effect of germination and extrusion on the phytic content of faba bean, Alonso et al. [71] reported 61% and 27% reductions due to germination and extrusion, respectively. In contrast, trypsin inhibitor was reduced to a much greater extent when seeds were extruded compared to when they were germinated [71]. Germination is therefore a viable method to be used in combination with other processing methods such as cooking and extrusion to reduce the antinutrients in seeds.

Soaking, which is usually conducted as part of the germination process, is traditionally used to remove antinutrients from foods and to reduce the cooking times of legumes. It has been reported to reduce phytic acid, condensed tannins, and protease inhibitors to varying extents. The changes depend on the plant sources and germination conditions. For example, the soaking of common bean and faba beans reduced phytic acid by 6% and 30%, and reduced the condensed tannins by 24% and 48%, respectively. Additionally, soaking did not change the activity of the protease inhibitors in the faba bean, while chymotrypsin and α-amylase inhibitors significantly declined in the common bean [71]. Similarly, different varieties of millets exhibited a wide range of reductions in phytic acid and condensed tannins in response to soaking [27]. In contrast to the observed reduction in antinutrients, the phytic acid levels in the red kidney bean did not change after soaking, while condensed tannins were reduced by 74%. The decrease in phytic acid and condensed tannins during soaking is attributed to the leaching of these components. Therefore, the seed structure can affect the extent to which these components are reduced. Moreover, condensed tannins are water-soluble and therefore generally exhibit a greater reduction compared to phytic acid during soaking [71]. Some seeds may also undergo minor enzymatic changes during soaking, which degrades phytic acid and some trypsin inhibitors.

In general, subsequent germination reduces the antinutrients to a much greater extent than soaking alone, due to the increased activity of enzymes that accompanies germination, such as the increased phytase activity which degrades phytic acid. The further reduction in condensed tannins is attributed to the formation of hydrophobic associations with proteins and enzymes. The reduction in antinutrients was reported to be strongly correlated with the germination time and temperature [73]. However, a reverse effect on trypsin inhibitor was observed when germination was prolonged and soybean hypocotyl length was allowed to grow to 5 cm, attributed to the photosynthetic resynthesis of the compound as the hypocotyl lengthened [77]. The levels of trypsin inhibitor also correlated with IVPD. Hence, it is important to stop the germination process at an optimal time to prevent the resynthesis of antinutrients that would affect the digestibility of the protein.

## 4. Priming Seeds to Improve Germination and Induce the Production of Secondary Metabolites

To further improve germination outcomes, particularly in changing environmental conditions, seeds can be “primed”, via either physical or chemical methods. Seed priming elicits a response that prepares the seed for environmental stresses, such as drought, high salinity, or temperature changes, via metabolic adaptations and the upregulation of cell defense mechanisms [81]. This can involve the mobilization of storage reserves, macronutrient synthesis, and the production of plant hormones and secondary metabolites [82]. Essentially, seed priming stimulates similar changes to those observed during germination, without reaching radical emergence. Primed seeds can then be dehydrated and stored if required but will be able to resume germination with improved responses to abiotic stressors. As it involves metabolic changes, priming can only be applied following imbibition, when the seed metabolism has resumed [82]. A range of priming techniques, including hydro-priming, osmo-priming, hormonal-priming, chemical priming, and bio-priming, have been found to improve the germination and growth performance of plants [82]. For example, the priming of wheat seeds with N-hexanoyl-L-homoserine lactone, a bacterial quorum-sensing signal, increased the germination percentage from 81% (in unprimed seeds) to 97% and increased the radicle length from 7 to 9.5 mm [83]. Both drought-tolerant and drought-sensitive rice seeds primed with methyl jasmonate, a plant stress hormone, were found to grow better in drought conditions than unprimed seeds [84]. Similarly, the priming of cucumber seeds with 5% potassium nitrate increased the germination percentage and rate (20% and 55%, respectively), reduced the mean germination time, and provided protection against reduced soil moisture [85].

In addition to the improvements in germination performance, seed priming can also be employed to increase the production of plant secondary metabolites that function as bioactives in humans. Secondary metabolites are substances synthesized for protection when plants are subjected to environmental stress, such as antioxidants, amino acids, and their derivatives [86]. The process of germination triggers the production of reactive oxygen species (ROS) and reactive nitrogen species, and enzymes and other metabolites (antioxidants) are subsequently produced to prevent cell damage. Other stresses during this point can also influence the production of plant metabolites with nutritional value. For example, it has been proposed that in response to wounding stress, ATP is released from damaged cells, which then binds to ATP receptors on other cells, stimulating the production of ROS, ethylene, or jasmonic acid, which function as signaling molecules [87]. This activates a stress response in plant cells, resulting in the production of compounds such as phenolics and glucosinolates, which are thought to provide antioxidant and anti-inflammatory effects to humans. Hence, there is much interest in enhancing the content of these compounds in germinated food products, allowing them to be used as functional foods [88].

Research on stimulating seeds to enhance the production of these metabolites has therefore been an ongoing agenda [89]. The treatment of broccoli or radish seeds with methyl jasmonate, a plant stress hormone, as a priming agent increased their contents of glucosinolates [90]. This is particularly important, as increased levels of glucosinolates have been reported to translate into clinical benefits; broccoli sprouts stimulated to produce more glucosinolates were found to reduce the expression of inflammatory markers when consumed raw (30 g/day for 10 weeks) by 40 patients with body mass indices of 24.9–29.9 [91]. Furthermore, the anti-inflammatory effects correlated with the presence of glucosinolate metabolites. A high salinity level of 200 mM of NaCl during the germination of wheat seedlings stimulated the production of a number of phenolic compounds compared to seedlings germinated in distilled water, with a total increase in all investigated compounds of up to 271% [92]. However, this was also accompanied by a significant reduction in the germination percentage as well as the lengths of the seedling roots and shoots. Seed priming can therefore be a useful tool to improve the germination performance or the nutritional quality of germinated seeds, but it may be difficult to simultaneously achieve improvements in both parameters.

## 5. PEF Treatments to Improve Seed Germination

Although germination is considered a green technology, it is still time-consuming. The long germination cycle and slow turn-over has made the process less appealing for industry adoption. Therefore, it is imperative that the process is shortened to save time without compromising the germination capacity and protein quality. Furthermore, despite the enhanced nutritional value of germinated seeds, there is clearly an added benefit if the targeted treatment can further enhance the contents of secondary metabolites. Thus, there is a growing interest in using novel technologies to break seed dormancy, improve germination performance, and enhance the nutritional quality of germinated seeds. PEF is an emerging non-thermal and chemical-free green technology that has demonstrated value in improving the nutritional and functional qualities of numerous food products. However, the optimal PEF settings required to achieve these effects may differ significantly depending on the sample in question and the desired outcome.

### 5.1. The Effects of PEF Treatments before and after Imbibition on Water Uptake and Germination Performance

**Figure 3:**
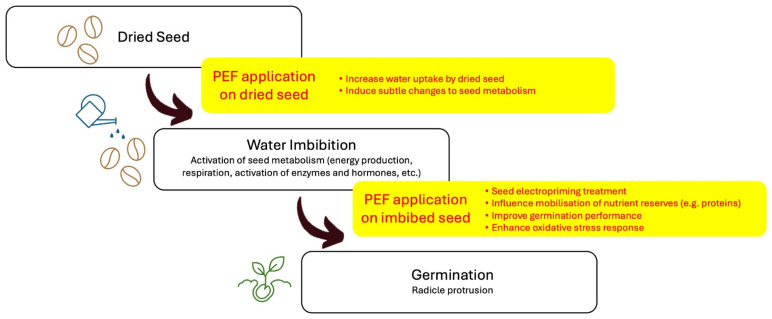
Potential applications of PEF to improve germination outcomes. PEF can be applied on seeds prior to imbibition, where the electroporation effect can enhance water uptake while also inducing slight metabolic changes, ultimately aiding the germination process. When applied after imbibition as a priming treatment, PEF can induce more drastic changes in the imbibed seed, providing a greater resistance towards future stressors and enhancing the nutritional value through the mobilization and synthesis of proteins and the production of secondary metabolites.

The electroporation effect induced by a PEF suggests it would enhance seed water uptake, thereby improving the efficiency of germination. However, the timing of PEF application (i.e., before or after seed imbibition) is an important consideration (Figure 3). A PEF treatment (3 kV/cm, 10 μs, and 100 or 200 pulses, equating to 9.9 or 19.8 kJ/kg) was applied to wheat seeds before or after the first of two soaking periods to explore the effectiveness of PEF at different moisture contents [93]. The PEF treatment (19.8 kJ/kg) prior to the first hydration cycle resulted in a 25% increase in the water uptake rate during the first minutes of soaking and an increase in water retention after the resting period. However, for the PEF treatment at a lower intensity (9.9 kJ/kg), a decrease in the moisture content was observed after the resting period. Similarly, wheat seeds PEF-treated before soaking at an electric field strength (EFS) less than 6 kV/cm, a 100 µs pulse width, and with 25 pulses, did not show an increased water uptake, but those treated at 6 kV/cm with 50 pulses exhibited an increased water content from 50.93% to 56.56% [94]. In contrast, PEF treatment applied after the first hydration (where seeds had ~40% moisture content) did not show an increased rate of water uptake but the water retention and the equilibrium moisture content were increased in response to both lower (9.9 kJ/kg) and higher (19.8 kJ/kg) PEF treatments [93]. The increase in the moisture content for seeds PEF-treated at a higher intensity can be explained by the reversible permeabilization of the seed that allows water to enter the cell and, at the same time, causes the buildup of osmotic forces that attracts water [93]. In addition, PEF treatment can cause an electrical coagulation of proteins and colloids that can increase water retention [95]. A charge is also created on the surface of the seeds that adsorbs water; however, a portion of this adsorbed water can be lost via evaporation. This could explain why seeds which were not presoaked before the PEF treatment at the lower intensity of 9.9 kJ/kg exhibited a decrease in their moisture content after the resting period. In contrast, the higher water retention exhibited by seeds PEF-treated at the same intensity, but which were presoaked prior to the PEF treatment, suggests that water was able to migrate inside the cells and was retained due to the mechanisms mentioned above. In this sense, PEF at the same intensity (9.9 kJ/kg) induced more intense effects on the seeds with a higher moisture content which can be related to the higher electrical conductivity of the sample during PEF treatment [93].

Given the importance of seed moisture content, most studies investigating the use of PEF to enhance the rate and efficiency of germination have applied PEF following an initial soaking period (Table 1). For example, a PEF treatment (5–15 kV/cm) of *Arabidopsis thaliana* improved the germination rate from 79% for untreated seeds to 87–99% after 14 days [96]. The greatest improvement in germination rate was observed in response to 10 kV/cm, corresponding to a specific pulse energy of 1000 J/kg, which increased the germination rate on day five from 67% to 94% and up to 99% on day fourteen. A similar pattern was observed regarding the leaf area, in which seeds stimulated with PEF at 10 kV/cm showed a greater increase in their leaf area at day 14 in comparison to untreated and all other PEF-treated seeds. Using a response surface methodology, Song et al. [97] selected three different PEF treatments to evaluate the effects on the germination potential of *Scutellaria baicalensis*. The germination percentage, rate, and index were the greatest compared to untreated seeds when treated with 99 pulses at a pulse intensity of 0.5 kV/cm and a pulse width of 120 µs. Furthermore, the mean germination time reduced from 1.94 (untreated) to 1.53 days. Similarly, the germination rate of wheat seeds after one day increased from 81% for control seeds to 95% for PEF (9.9 kJ/kg)-treated wheat seeds [93]. After 3 days, the germination capacity plateaued at 97% and was not significantly different to the controls. Nevertheless, this study demonstrated that PEF has the potential to reduce germination time, ultimately making the process more efficient [93]. In contrast, the germination performance was reduced for barley seeds subjected to a low PEF intensity (1.2 kV/cm, 1 ms, 50 pulses, 0.91 kJ/kg) after soaking. Similarly, PEF at an intensity of 0.3–0.7 kV/cm (with an equivalent specific energy input of 0.5–3.0 kJ/kg) applied to pre-soaked faba bean seeds did not improve their germination rate, germination percentage, or radicle length after 72 h of germination [98]. Qu et al. [99] reported that although PEF (1 kV/cm, 120 Hz, 1 h) did not alter the germination rate of buckwheat seeds, it did increase the root length by 18%. The intensity of the PEF process parameters applied determines the treatment’s effect on the germination performance across seeds. Some sources mentioned that the low to mild treatment intensities, which induce the reversible permeabilization of the cells, are those with an EFS not exceeding 1.5 kV/cm and an energy input not exceeding 5 kJ/kg [21,22]. However, studies on PEF applications for seed germination have reported higher electric field intensities (up to 6 kV/cm) and specific energy inputs (up to 19.8 kJ/kg) that have positively impacted germination rates and performances [93,94]. This could be due to other PEF-related factors (the equipment design, other processing parameters) or sample factors such as the structural toughness or the electrical conductivity of the seeds. As mentioned above, presoaking the seeds prior to the PEF treatment would enhance its effectiveness due to the enhanced electrical conductivity. Therefore, in the application of PEF on seed germination, it is important to strike a balance between the PEF processing parameters and the sample properties, such as the moisture content, to affect the seed positively.

### 5.2. Effects of Electropriming Using PEF on Secondary Metabolites in Germinated Seeds

In addition to improving germination efficiency, priming using PEF may improve the nutritional values of seeds, usually achieved by inducing a stress response. Biological, chemical, or physical factors have been used to elicit stress to induce the production of phytochemicals in seeds. While these can be very effective, they can also adversely affect the physiological properties of seeds, they may incur added costs from separating the elicitors from the desired compounds, and they are sometimes toxic [103]. Hence, physical priming treatments such as the use of PEF have more recently been investigated as a greener and cleaner method of producing plant secondary metabolites [104].

The induction of secondary metabolite production in response to PEF has been clearly demonstrated in whole plants or plant cell cultures. For example, PEF treatment (1.6 kV/cm, 0.32 J/kg) of a grape cell culture increased the anthocyanin content by ~20% after one day [105]. After 14 days, the growth rate of the PEF-treated grape cells had reduced slightly (~7%) compared to that of the untreated grapes, but the anthocyanin content had increased by more than 50%. Similarly, the treatment of soy root suspension culture cells with PEF at 1.6 kV/cm resulted in higher concentrations of genistein and daidzein than PEF at 1.2 kV/cm or 2–2.4 kV/cm [88]. Comparatively few papers have investigated the use of PEF to enhance secondary metabolite production in seeds. Those that have done so, however, have observed favorable results. PEF treatment (0.5 kV/cm, 120 µs pulse width, and 99 pulses), selected to optimize germination, was found to nearly double the soluble sugar content and increase α-amylase activity by ~40% compared to untreated *Scutellaria baicalensis* seeds on days 10 and 15 [97]. The soluble protein content also increased compared to that of the control by 77, 69, and 41% on days 5, 10, and 15, respectively. Leong et al. [100] found that PEF treatment at 2 kV/cm (but not 0.5 or 1.4 kV/cm) increased the levels of glutathione and ascorbate and increased the activity of the antioxidant enzymes catalase, glutathione reductase, glutathione peroxidase, superoxide dismutase and ascorbate peroxidase by 70–100% in 7-day-old wheatgrass seedlings [100]. PEF induced the production of these components which comprise the ascorbate–glutathione pathway (Asada–Halliwell pathway), the antioxidant defense mechanism in plants that mainly detoxifies the hydrogen peroxide in a plant cell [106]. The resulting digests of these seedlings provided a protective effect to Caco-2 cells, with an ~30% increase in Caco-2 cell viability observed in response to H_2_O_2_ (500 µM for 1 h) treatment. Importantly, the PEF-induced increase in antioxidant enzyme activity was most pronounced at seed water contents of 45–50%. Increasing the moisture of seeds before applying PEF effectively increases their electrical conductivity and consequently enhances the effect of the treatment, further highlighting the significance of the moisture content in determining the effectiveness of a PEF treatment. Additionally, PEF could be used in combination with chemical elicitors. For example, the application of both PEF (2.5–5 kV/cm, 1–2 pulses) and jasmonic acid (50 µM) to hairy root cultures of rocket was more effective in enhancing the glucosinolate content than either treatment alone [107]. This opens up the potential uses of PEF, as it could be used to either replace or enhance the effect of chemical elicitors.

It is important to note, however, that the PEF parameters used to optimize secondary metabolite production may come at the cost of a reduced germination performance. For example, while an EFS of 2 kV/cm was found to significantly enhance the production of antioxidants in wheatgrass seedlings, this was also associated with a reduction in the height of both the coleoptiles and the seedlings compared to those of untreated seedlings [100]. This is not surprising, given the cell stress induced by PEF during electropriming. It does, however, highlight the importance of assessing multiple outcomes of PEF-induced electropriming. Future investigations should therefore consider secondary metabolite production when using PEF as a priming tool to better understand the complex effects of this technology.

While there are several studies that focus on the effect of the PEF-induced accumulation of antioxidants in plant systems, only one study has investigated the effect on protein quality and digestibility following electropriming with PEF. Johnston et al. [98] reported an increase in the protein content of the faba bean flour made from seeds that had been PEF-treated at 0.3–0.5 kV/cm, then germinated for 72 h, compared to that of non-germinated seeds. However, the values obtained from combining PEF and germination were not significantly different compared to germination alone, and the use of PEF alone did not substantially change the protein content of the ungerminated seeds. This suggests that germination had a greater impact on protein content than did the PEF treatment. Further studies to elucidate the mechanisms through which PEF and germination act together to affect protein contents and quality are needed [98]. It is important to note that although no changes in the crude protein content were observed, the protein composition in germinating seeds may change in response to PEF. Environmental stresses trigger a metabolic adaptation in plants that leads to the accumulation of specific amino acids and secondary metabolites from amino acid metabolism. Highly abundant amino acids like proline, arginine, asparagine, glutamine, and γ-aminobutyric acid are synthesized to combat osmotic stress, and one study did report a 2-fold increase in the proline content of *Scutellaria baicalensis* seeds 10 days following a PEF treatment selected to optimize germination [97]. Non-abundant amino acids are accumulated due to the increased protein turnover and are degraded [108], and non-protein amino acids are also produced as secondary metabolites [109]. In addition, protein hydrolysis increases in response to several abiotic stressors to provide amino acids as substrates for ATP production and to remobilize reduced nitrogen and sulfur [110]. This, in turn, results in an increase in the amounts of free amino acids. Branched chain and aromatic amino acids as well as lysine show high fold increases under different stress conditions [108]. Although such responses have predominantly been observed in plants at a different developmental phase than germinating seeds, there are similarities in their coping mechanisms. In this case, the use of PEF has the potential to change the amino acid composition of germinated seeds.

In the aforementioned study on faba beans [98], the PEF treatment enhanced the starch and protein digestibility of the faba beans. When flour from PEF-treated and germinated seeds was used to substitute 30% of the wheat flour in bread, an improvement in the IVPD in the gastric stage, but not in the intestinal phase, was observed. PEF treatment may enhance the activity of enzymes during germination that could impact the starch and protein digestibility of germinated seeds, further enhancing their nutritional value. Given that protein–starch interactions tend to hinder protein digestibility, this could suggest that protein digestibility might improve, but the increased digestibility of starch could also be a disadvantage if it resulted in increased blood glucose levels following consumption. The presence of antinutrients also influences protein digestibility. A low intensity PEF treatment (0.3–0.7 kV/cm) increased the phytic acid content of faba bean, which was attributed to the seeds’ response to oxidative stress [98]. It should be noted that after germinating the seeds for 72 h, a reduction in the phytic acid content was observed for PEF-treated seeds. This demonstrates the complexity of PEF and germination treatments in altering the protein digestibility of seeds. More studies are needed to validate and elucidate the effect of PEF and germination on the protein quality and overall nutritional value of seeds.

It is worth noting that the presence of increased ROS caused by PEF may promote protein oxidation. Zhang et al. [111] reported protein oxidation in germinated rice grains and were able to quantify 288 carbonylated peptides corresponding to 144 proteins. Of these carbonylated proteins, 66 were involved in maintaining the levels of ROS. Protein carbonylation occurs through the direct oxidation of side chains of lysine, arginine, proline, and threonine residues. Protein carbonylation may result in the misfolding, loss of function, degradation, and aggregation of proteins that are toxic to cells, therefore impairing protein functionality and reducing protein digestibility. In muscle proteins, protein carbonylation was linked to deterioration in the gelling, emulsifying, and water-holding capacities of the proteins [112]. However, mild protein oxidation may facilitate protein functionality and has been reported to produce proteins with stable gels [113]. More recently, Vahalová et al. [114] demonstrated, using a biochemiluminescence-sensing system, that PEF treatment induced ROS production. Furthermore, when bovine serum albumin was added to the system as a model protein, the bioluminescence increased, suggesting that carbonylation had occurred. Such findings have yet to be confirmed in plant cells, though it would be reasonable to assume a threshold for beneficial PEF effects, after which the damage to the cell becomes too great to outweigh the increased presence of nutritional compounds.

## 6. Future Research and Challenges Associated with Using PEF for Seed Germination

The research into the potential of PEF to improve germination-related outcomes is still an area in its infancy. The key challenges associated with PEF center on striking a balance between a stress response that is sufficient to induce the desired changes but does not reduce seed viability, and, as outlined above, the fact that different seeds can show varying responses to the same PEF treatment settings. Furthermore, important changes in the seed, such as secondary metabolite production or changes in protein solubility, may be occurring despite a lack of improved germination performance. While most studies investigating the effect of the use of PEF on seeds, to date, have focused on germination performance or post-germination growth, future studies should also consider the impact of PEF on seed nutrients.

The incorporation of PEF into other stages of the germination process is still yet to be thoroughly explored. For example, PEF has often been used to inactivate microorganisms (albeit with little to no effect on spore inactivation) via the irreversible electroporation of the cell membrane. However, the use of PEF has only recently been studied as a potential technology for seed microbial inactivation. Evrendilek et al. [115] treated various microbial-contaminated seeds, including wheat, barley, parsley, onion, and lettuce, with PEF using an EFS of 12 kV/cm at an energy of 240 or 960 J. The effectiveness of PEF at inactivating microbes varied between seeds, reducing as little as 36–37% of the bacteria on parsley and lettuce seeds at 240 J (48–49% at 960 J), and greater than 99% of the bacteria on winter wheat, tomato, and rocket seeds at 960 J. In contrast, 7–10% of mold and yeast was inactivated on rocket, while up to 98% was inactivated on winter wheat and parsley. Importantly, the germination rate of all seeds treated with 960 J increased by 2–15%, highlighting that the overall outcome of the PEF treatment was beneficial. A subsequent study on wheat seeds identified a much lower energy (6.1 J, with a frequency of 161.8 Hz) as being optimal to enhance germination and increase stress tolerance, while also inactivating microbes [116]. Additionally, no study has investigated the effect of PEF treatments on seed viability in relation to storage time. This is important in cases where PEF-treated seeds cannot be germinated immediately or if they are to be sold or distributed. In this context, the moisture control, the drying processes, and the storage conditions are critical factors that need to be investigated.

While PEF treatment has demonstrated its potential as a tool for enhancing seed germination, it is important to consider its economic feasibility. The cost of an industrial PEF system can vary depending on its capacity and intended application. For example, a 200 kW PEF system can cost between 300 and 500 thousand USD [117]. This estimate does not include ancillary equipment. In addition, the cost of the electricity for the overall lifetime of using the equipment may be higher than the cost of the equipment itself. However, considering the depreciation cost of the equipment, which can last 10–20 years, and the electricity cost, PEF could reduce the cost of producing a product by 30–90% (compared to heating) in peeling or extraction applications [117]. While non-thermal pasteurization using PEF can cost twice as much as heating, it is still only 10–20% of the cost of high-pressure processing (HPP), currently the most commonly used non-thermal pasteurization method, per liter of product [117,118]. Importantly, the cost of PEF treatment for seed germination (for example as an electropriming treatment) is expected to be less than that for pasteurization or tissue modification, as a significantly lower intensity of process parameters is required. The electricity usage for seed applications with PEF equipment is typically minimal, and the associated cost depends on the electricity generation technology employed. Recently, many food companies have transitioned to utilizing renewable energy sources, such as solar or wind power, for their electricity needs. This sustainable approach can also be considered for operating a PEF system effectively. Furthermore, the cost of commercial PEF systems can be reduced over time as the market for PEF systems grows [117].

## 7. Conclusions

The ever-increasing demand to produce nutritious food in changing environmental conditions necessitates the utilization of novel technologies to enhance seed germination. PEF treatment has been well-documented to improve the germination performance of seeds, both via its effect on water uptake and priming the seed for growth. Furthermore, the seed moisture content during PEF application has been reported to influence the effectiveness of the treatment, with increases in moisture content enhancing the effect of PEF treatment. More recent research has demonstrated the potential of PEF to enhance the nutritional value of germinated seeds by increasing the production of protein and secondary metabolites and increasing the IVPD. The specific PEF treatment protocol will be dictated by the desired outcome (i.e., improving germination rates to make food production more efficient versus increasing the protein or secondary metabolite contents of germinated foods). In doing so, however, one must also consider the potential disadvantages, such as the resynthesis of antinutritional compounds, the increased starch digestibility, and the protein oxidation, and a balance needs to be reached to maximize nutritional value.

## Figures and Tables

**Table 1:** The effects of PEF on the moisture and germination performance of seeds.

Plant Type	Soaking Parameters	Pulsed Electric Field (PEF) Parameters	Highlights on Moisture and Germination Observations	Reference
Faba bean	20 °C, 18 h	EFS: 0.3 kV/cm, 0.5 kV/cm, 0.7 kV/cm NP: 1000 PW: 20 µs PS: bipolar fixed square waves PF: 20 Hz PM: distilled water SPI: 0.5, 1.0–1.5, 2.5–3.0 kJ/kg Equipment: ELCRACK-HVP 5 TCDs: 10 × 80 × 50 cm ED: 8 cm Application timing: After soaking	The PEF treatment had no significant effect on the germination rate, germination percentage, or radicle length of the seeds after 72 h of germination. The PEF treatment at 0.3 kV/cm increased the mean radicle length of the seeds within 24 h of germination. The PEF treatment at 0.7 kV/cm had a negative impact on germination rate and radicle length of the seeds.	[98]
Wheat	18 °C, total Soaking cycle: 4 h 1st soaking, 15 h resting period, 3 h 2nd soaking	EFS: 3 kV/cm NP: 100, 200 PW: 10 µs PS: monopolar square pulses PF: 10 Hz PM: tap water SPI: 9.9, 19.8 kJ/kg ED: 1.7 cm Equipment: pulsus PM1–10 Treatment chamber dimensions TCDs: 10 × 10 cm Application timing: Before or after soaking	The hydration rate of the seeds was enhanced when PEF was applied before hydration, but a lower PEF intensity was needed to increase water retention when it was applied after hydration. The germination capacity of the seeds after 1 d was increased by 16% for a PEF treatment performed at a lower intensity after hydration; however, PEF at a higher intensity before hydration increased the radicle length of the seeds germinated after 3 d. The moisture content of the seeds influenced the effectiveness of the PEF treatments.	[93]
Wheat, *Tritium aestivum* L.	3, 6, 10, 24 h 20 ± 2 °C	EFS: 2, 4, 6 kV/cm NP: 25, 50 PW: 100 µs PF: 1 Hz PM: distilled water SPI: 1.5, 2.5, 3.7, 7.5 kJ/kg Equipment: laboratory-scale PEF (EX-1900, SCUT, PEF-Team) TCDs: 10 × 11 cm Application timing: Before soaking	PEF at an EFS = 6 kV/cm and 50 pulses increased the water uptake from 50.93% to 56.56% after 24 h. The water uptake increased as the PEF intensity and number of pulses increased. The germination rate increased with PEF intensity.	[94]
*Arabidopsis thaliana*		EFS: 5, 10, 15, 20 kV/cm NP: 100 PW: 10 ns PF: 5 Hz PM: 1% sodium chloride solution SPI: 0.25, 1, 2.25, 4 kJ/kg Equipment: SL300 high voltage DC source with 100 MΩ capacitor Application timing: After soaking	PEF at an SPI = ~ 1 kJ/kg was optimal for increasing the growth rate and germination percentage of the seeds. PEF at an EFS of 5–15 kV/cm increased the germination rate from 79% for untreated seeds to 87–99% for PEF-treated seeds.	[96]
*Scutellaria baicalensis*	12 h 40 °C	EFS: 0.5, 0.5, 1.705 kV/cm NP: 28, 60, 99 PW: 120, 40, 59 PM: distilled water Equipment: BTX ECM830 Square Save Electroporation System (BTX) TCDs: 20 × 20 mm Application timing: after soaking	The germination percentage increased and the mean germination time decreased for all three PEF treatments. The electrical conductivity was reduced for 48 h post-PEF treatment.	[97]
Buckwheat	Not reported.	EFS: 1 kV/cm PF: 120 Hz Treatment time: 1 h TCDs: 40 × 60 cm	No significant changes were seen in the sprouting rate or germination index between the PEF-treated and control seeds. The root length post-germination increased by 18% for PEF-treated seeds.	[99]
Wheat, *Triticum aestivum* L.	24 h, 20 °C	EFS: 0.5, 1.4, 2.0 kV/cm NP: 100 PW: 20 µs PS: bipolar fixed square waves PF: 5 Hz PM: distilled water SPI: 0.26 ± 0.01, 0.71 ± 0.01, 1.54 ± 0.03 kJ/kg Equipment: ELCRACK-HVP 5 TCDs: 10 × 80 × 50 cm ED: 8 cm Application timing: After soaking	The seedling size increased at an EFS of 1.4 kV/cm, but decreased at an EFS of 2 kV/Cm. The PEF treatment at an EFS of 2 kV/cm reduced the coleoptile and primary leaf growth. The EFS can influence seedling size and growth performance.	[100]
Barley Voyager variety	28 h, 21 °C, aerated	EFS: 1.0, 3.0 kV/cm PW: 20 µs PS: bipolar fixed square waves PF: 20 Hz PM: distilled water SPI: 14.0, 20.0 kJ/kg ED: 8 (800 mL) or 24 (9.6 L) TCDs: 800 mL or 9.6 L cell Equipment: Elea PEF-Pilot Dual system Application time: After soaking for 2 min	PEF treatment increased the rootlet length of the germinated seed. PEF treatment increased the germination rate by 2.9%. The fastest rate of germination was achieved with PEF at 1.0 kV/cm and 14.0 kJ/kg. PEF did not affect the moisture content of the seeds.	[101]
Barley, *Hordeum vulgare* cv. Prestige	24 h, 20 °C, Soaking cycle: 6 h soaking followed by 12 h rest period	EFS: 0.275, 0.40, 0.60, 0.80, 1.0, 1.2 kV/cm NP: 50 PW: 1 ms PS: rectangular SPI: 0.048, 0.1, 0.227, 0.405, 0.630, 0.910 kJ/kg Equipment: CEPT pulse generator ED: 0.4 cm Application timing: After soaking	PEF had no significant effect on the gross metabolic activity for 22 h after PEF treatment. There was a significant reduction in radicle elongation at an EFS of 1.2 kV/cm.	[102]

Electric field strength (EFS). Number of pulses (NP). Pulse width (PW). Pulse shape (PS). Pulse frequency (PF). Pulsing medium (PM). Specific energy input (SPI). Electrode distance (ED). Treatment chamber dimensions (TCDs).

## Data Availability

No new data were created or analyzed in this study. Data sharing is not applicable to this article.

## Supplementary Materials

The following supporting information can be downloaded at: https://www.mdpi.com/article/10.3390/foods13111598/s1, Table S1: Combinations of sterilization, soaking, and sprouting conditions used to germinate seeds, Table S2: Changes in the protein content and composition of germinated seeds.
