# Supplementary material for: Effect of Germination on Seed Protein Quality and Secondary Metabolites and Potential Modulation by Pulsed Electric Field Treatment
Source: Foods. 2024 May 21;13(11):1598. doi: 10.3390/foods13111598 (PMC11172214; doi:10.3390/foods13111598)
Supplement: Supplementary file 1 [file foods-13-01598-s001.zip › foods-2985234-supplementary.pdf]

Supplementary Material

Effect of germination on seed protein quality and secondary metabolites and potential modulation by pulsed electric field treatment

Norma Cecille Bagarinao [1,2], Jessie King [1,2], Sze Ying Leong [1,2], Dominic Agyei [1], Kevin Sutton [2,3] and Indrawati Oey [1,2,*]

[1] Department of Food Science, University of Otago, PO Box 56, Dunedin 9054, New Zealand
[2] Riddet Institute, Private Bag 11 222, Palmerston North 4442, New Zealand
[3] The New Zealand Institute for Plant & Food Research Limited, Private Bag 4704, Christchurch Mail Centre, Christchurch 8140, New Zealand
* Correspondence: indrawati.oey@otago.ac.nz

Table S1. Combinations of sterilization, soaking, and sprouting conditions used to germinate seeds.

Seed	Sterilization	Soaking	Sprouting	Reference
Oats, hull-less	0.2% formaldehyde solution*	Overnight	1-8 days, 20 °C	[1]
Oats	NR	24 ± 1 h, endpoint moisture content of 45%, wet and dry phase cycle changing every 3 h for a total of 7 cycles	2-6 days, 10 °C - 20 °C	[2]
Oats, hull-less	1% sodium hypochlorite solution, 20 s	12 h, 25 °C, 1:5 w/v SWR, aeration for 1 h every 4 h	0.5 – 3 days, 25 °C, 95% RH	[3]
Sorghum	0.2% formaldehyde solution*	Overnight, 1:3 w/v SWR	1 - 9 days; 20, 25 and 23 °C	[4]
Sorghum	NR	20 h, 1:5 w/v SWR	3 days, room temperature	[5]
Barley, rye, and wheat (hull-less)	NR	24 ± 1 h, 22 ± 2 °C, 1:2 w/v SWR	0.5 – 2 days, 35 °C, 95 ± 2%, dark	[6]
Millets	0.1% sodium hypochlorite, 30 min	18 h, ~25 °C, 1:4 w/v SWR, water changed every 2 h	0.5 – 2 days, 25 °C, 85% RH, watered when necessary	[7]
Triticale	0.2% formaldehyde solution*	Overnight	1-8 days, 20 °C, dark	[8]
Amaranth	NR	24 h, 1:5 w/v SWR	1 – 3 days, 22, 26, 30 °C	[9]
Buckwheat	NR	24 ± 1 h, endpoint moisture content: 45%, wet and dry phase cycle: 3 h, 4 cycles	2- 6 days, 10 °C to 20 °C	[2]
Soybean	70% ethanol, 1 min	14 h, 25 °C, 1:4 w/v SWR	25 °C, 80% RH, endpoint: hypocotyl length at 0, 1, 3, or 5 cm	[10]
Soybean	7% sodium hypochlorite solution, 1 min	8 h	~ 7 days, 21 °C, 12 h light/dark cycle, watered daily	[11]
Chickpea	7% sodium hypochlorite solution, 30 min	12 h, 1:5 w/v SWR	2 days, 30 °C	[12]
Chickpea	NR	10 - 60 °C, until equilibrium moisture was reached	4 days, 28 ± 1 °C	[13]
Chickpea	7% sodium hypochlorite solution, 30 min	5.5 h, 1:4 w/v SWR, shaking every 30 min	6 days at 25 °C, 99% RH, dark, 50% seed moisture content	[14]
Yellow Pea	0.07% (w/v) sodium hypochlorite, 30 min	Overnight, ~22 °C	1 – 3 days, room temperature, dark, sprayed with water once a day	[15]
Fava bean and common bean	10% mercuric chloride solution	12 h, 30 °C, 1:5 w/v SWR	1 – 3 days, 25 °C, aired and dark, sprinkled with autoclaved, bi-deionized water with 0.01% sodium azide	[16]
Mung bean	Soaked in water at 80 °C for 5 min	4 h, 45 °C	0.5 – 3 days, 25 °C, showered with water every 3 h for 5 s,	[17]

Seed	Sterilization	Soaking	Sprouting	Reference
			ozone removed every 12 h for 20 min	
Sesame	NR	NR	0 – 4 days, 25 °C, dark, watered every 8 h	[18]
Hemp	1% (v/v) sodium hypochlorite, 10 min, with agitation	24 h, ~22 °C, dark	0 – 5 days, room temperature, dark, sprayed with 0.3% v/v hydrogen peroxide daily	[19]

* Sterilization was done after soaking.

Not reported (NR). Seed-to-water ratio (SWR). Relative humidity (RH).

Table S2. Changes in protein content and composition of germinated seeds.

Plant type	Germination conditions	% change in protein content after germination	Effect on protein fractions	Effect on amino acids	Reference
Terra oat grains, hull-less	8 days, 23 °C, daylight	+8.79	Lysine-rich fractions (non-protein nitrogen, albumin, and residue nitrogen) increased.<br><br>Lysine-poor fractions (globulin and prolamin) decreased	Thr, Ile, Leu, Lys increased.<br><br>Phe+Tyr, Val, Met+Cys decreased.	[1]
Oats, Avena nuda L.	6 days, 16 °C, dark	+16.02	Free amino acids increased.	All EAA and TAA increased.	[20]
Dehulled oat seeds (var. Meeri)	4 days, 18 °C, 90% RH, darkness	+11.42	45-75 kDa (globulin 3S and 7S) increased.	No significant change in the total EAA, but TNEAA and TAA decreased by 17% and 15%, respectively.	[21]
Oats	2 days, 35 °C, 95 ± 2%, dark	=	Soluble nitrogen and free amino nitrogen increased.<br><br>6 kDa, 20-27 kDa, and 40-50 kDa fractions decreased.	NR	[2]
Oats	2 days, 20 °C	NR	NR	All EAA and NEAA increased.	[22]
Naked oats, Baiyan II cultivar	3 days, 25 °C, 95 % RH	+ 10.4	NR	All EAA and NEAA increased.	[3]
Sorghum (*Sorghum bicolor* L. Moench)	3 days, room temperature	- 2.89	No significant change in albumin, globulin, kafirin, cross-linked kafirin and cross-linked glutelin fractions.<br><br>Free amino acids increased.	Most EAA and NEAA decreased except for Phe, Val, Ala, and Glu.	[5]
Sorghum	9 days, 22 °C with daylight	+34.0	Albumin increased.<br><br>Kafirin and cross-linked kafirin decreased.	TEAA increased after 3 days but decreased after 10 days of germination.	[4]
Wheat (*Triticum aestivum* L. var. Anzunbaengi)	1.92 days, 17.6 °C	+20.12	NR	TAA increased by 97% and the TEAA increased 5-fold.	[23]
Triticale (cross between wheat and rye)	8 days, 20 °C, dark	+15.2	Non-protein nitrogen increased.<br><br>Prolamin and glutelin decreased.	TEAA increased.	[8]
Amaranth (*Amaranth caudatus* L.)	0.75 days 30 °C	+11	60 kDa fractions decreased.<br><br>No significant changes in 34 kDa fractions.<br><br>22 and 16 kDa fractions decreased.	TEE increased after 18 h but decreased after 24 h of germination.<br><br>Proline increased 5-fold on day 2.	[24]
Chickpea cultivars	2 days, 30 °C	+ 2.92 - 3.11	11-19 kDa fractions increased.<br><br>25-100 kDa fractions decreased.	NR	[12]

Plant type	Germination conditions	% change in protein content after germination	Effect on protein fractions	Effect on amino acids	Reference
Pea (*Pisum sativum* L. variant Arvense cv. Esla)	6 days, 25 °C, 99% RH, dark	+11.14	Insoluble nitrogen and soluble protein nitrogen decreased.<br><br>Soluble non-protein nitrogen increased.<br><br>70 kDa (convicilin), 39-40 kDa (α-legumin), 12-30 to 35 kDa (vicilin), 20-25 kDa (β-legumin) fractions decreased.	NR	[25]
Pigeon pea (*Cajanus cajan*)	3 days, 28 °C	+17.66	NR	TAA, TEAA, and TNEAA increased.	[26]
Mung bean (*Vigna radiata*)	0.5 days	+9.76	NR	TAA and TEAA increased, but TNEAA decreased.	[27]
Soybean (*Glycine max* L. var. Woodworth)	5 days, 20-35 °C, light and dark, tap water	+10.6	NR	TAA and TEAA increased, but TNEAA decreased.	[28]
Sesame	4 days, 25 °C, dark	NR	20 kDa fractions increased.<br><br>35 and 40 kDa fractions decreased.	NR	[18]
Hemp	3 days, room temperature, dark	+1.81	10-20 kDa fractions increased.<br><br>Vicilin protein band (47 kDa) and edestin protein band (33 kDa) decreased.	NR	[19]

(+) statistically significant increase, (-) statistically significant decrease, (=) not statistically significant difference, Relative humidity (RH). Total amino acids (TAA). Essential amino acids (EAA). Non-essential amino acids (NEAA). Total EAA (TEAA). Total NEAA (TNEAA). Not reported (NR).

References

1. Wu, Y. Effect of germination on oats and oat protein. *Cereal Chemistry* **1983**, *60*, 418-420.
2. Hübner, F.; Arendt, E.K. Studies on the Influence of Germination Conditions on Protein Breakdown in Buckwheat and Oats. *Journal of the Institute of Brewing* **2010**, *116*, 3-13, doi:10.1002/j.2050-0416.2010.tb00392.x.
3. Li, Q.; Xu, J.-G. Changes in nutritive value and in vitro digestibility of proteins from naked oats during germination. *European Journal of Food Science and Technology* **2015**, *3*, 49-57.
4. Wu, Y.V.; Wall, J.S. Lysine content of protein increased by germination of normal and high-lysine sorghums. *Journal of Agricultural and Food Chemistry* **1980**, *28*, 455-458, doi:10.1021/jf60228a046.
5. Afify, A.E.-M.M.R.; El-Beltagi, H.S.; Abd El-Salam, S.M.; Omran, A.A. Protein solubility, digestibility and fractionation after germination of sorghum varieties. *PLoS One* **2012**, *7*, e31154, doi:10.1371/journal.pone.0031154.
6. Senhofa, S.; Kince, T.; Galoburda, R.; Cinkmanis, I.; Sabovics, M.; Sturite, I. Effects of germination on chemical composition of hull -less spring cereals. In Proceedings of the Research for Rural Development, 2016; pp. 91-97.
7. Sharma, B.; Gujral, H.S. Modifying the dough mixing behavior, protein & starch digestibility and antinutritional profile of minor millets by sprouting. *International Journal of Biological Macromolecules* **2020**, *153*, 962-970, doi:10.1016/j.ijbiomac.2019.10.225.
8. Wu, Y.V. Lysine content of triticale protein increased by germination. *Journal of Agricultural and Food Chemistry* **1982**, *30*, 820-823, doi:10.1021/jf00113a005.
9. Najdi Hejazi, S.; Orsat, V.; Azadi, B.; Kubow, S. Improvement of the in vitro protein digestibility of amaranth grain through optimization of the malting process. *Journal of Cereal Science* **2016**, *68*, 59-65, doi:10.1016/j.jcs.2015.11.007.
10. Aijie, L.; Shouwei, Y.; Li, L. Structure, trypsin inhibitor activity and functional properties of germinated soybean protein isolate. *International Journal of Food Science & Technology* **2014**, *49*, 911-919, doi:10.1111/ijfs.12386.
11. Bueno, D.B.; da Silva Júnior, S.I.; Seriani Chiarotto, A.B.; Cardoso, T.M.; Neto, J.A.; Lopes dos Reis, G.C.; Glória, M.B.A.; Tavano, O.L. The germination of soybeans increases the water-soluble components and could generate innovations in soy-based foods. *LWT* **2020**, *117*, 108599, doi:10.1016/j.lwt.2019.108599.
12. Sofi, S.A.; Singh, J.; Muzaffar, K.; Majid, D.; Dar, B.N. Physicochemical characteristics of protein isolates from native and germinated chickpea cultivars and their noodle quality. *International Journal of Gastronomy and Food Science* **2020**, *22*, 100258, doi:10.1016/j.ijgfs.2020.100258.
13. Kaur, R.; Prasad, K. Elucidation of temperature dependent hydration behaviour of chickpea seeds: Prerequisite for germination. *Biocatalysis and Agricultural Biotechnology* **2023**, *50*, 102669, doi:10.1016/j.bcab.2023.102669.
14. Xu, M.; Jin, Z.; Simsek, S.; Hall, C.; Rao, J.; Chen, B. Effect of germination on the chemical composition, thermal, pasting, and moisture sorption properties of flours from chickpea, lentil, and yellow pea. *Food Chemistry* **2019**, *295*, 579-587, doi:10.1016/j.foodchem.2019.05.167.
15. Setia, R.; Dai, Z.; Nickerson, M.T.; Sopiwnyk, E.; Malcolmson, L.; Ai, Y. Impacts of short-term germination on the chemical compositions, technological characteristics and nutritional quality of yellow pea and faba bean flours. *Food Research International* **2019**, *122*, 263-272, doi:10.1016/j.foodres.2019.04.021.
16. Alonso, R.; Aguirre, A.; Marzo, F. Effects of extrusion and traditional processing methods on antinutrients and in vitro digestibility of protein and starch in faba and kidney beans. *Food Chemistry* **2000**, *68*, 159-165, doi:10.1016/S0308-8146(99)00169-7.
17. Liu, Y.; Xu, M.; Wu, H.; Jing, L.; Gong, B.; Gou, M.; Zhao, K.; Li, W. The compositional, physicochemical and functional properties of germinated mung bean flour and its addition on quality of wheat flour noodle. *Journal of Food Science and Technology* **2018**, *55*, 5142-5152, doi:10.1007/s13197-018-3460-z.

18. Di, Y.; Li, X.; Chang, X.; Gu, R.; Duan, X.; Liu, F.; Liu, X.; Wang, Y. Impact of germination on structural, functional properties and in vitro protein digestibility of sesame (Sesamum indicum L.) protein. *LWT* **2022**, *154*, 112651, doi:10.1016/j.lwt.2021.112651.
19. Liu, M.; Childs, M.; Loos, M.; Taylor, A.; Smart, L.B.; Abbaspourrad, A. The effects of germination on the composition and functional properties of hemp seed protein isolate. *Food Hydrocolloids* **2023**, *134*, 108085, doi:10.1016/j.foodhyd.2022.108085.
20. Tian, B.; Xie, B.; Shi, J.; Wu, J.; Cai, Y.; Xu, T.; Xue, S.; Deng, Q. Physicochemical changes of oat seeds during germination. *Food Chemistry* **2010**, *119*, 1195-1200, doi:10.1016/j.foodchem.2009.08.035.
21. Aparicio-García, N.; Martínez-Villaluenga, C.; Frias, J.; Peñas, E. Sprouted oat as a potential gluten-free ingredient with enhanced nutritional and bioactive properties. *Food Chemistry* **2021**, *338*, 127972, doi:10.1016/j.foodchem.2020.127972.
22. Tang, S.; Mao, G.; Yuan, Y.; Weng, Y.; Zhu, R.; Cai, C.; Mao, J. Optimization of oat seed steeping and germination temperatures to maximize nutrient content and antioxidant activity. *Journal of Food Processing and Preservation* **2020**, *44*, e14683, doi:10.1111/jfpp.14683.
23. Aung, T.; Kim, B.R.; Kim, S.; Shin, E.-C.; Kim, M.J. Comparative volatiles, amino acids, and phenolic compounds and characteristics of roasted germinated wheat (Triticum aestivum L.) during beverage preparation. *LWT* **2023**, *173*, 114412, doi:10.1016/j.lwt.2022.114412.
24. Guardianelli, L.M.; Salinas, M.V.; Puppo, M.C. Chemical and thermal properties of flours from germinated amaranth seeds. *Journal of Food Measurement and Characterization* **2019**, *13*, 1078-1088, doi:10.1007/s11694-018-00023-1.
25. Urbano, G.; López-Jurado, M.; Frejnagel, S.ł.; Gómez-Villalva, E.; Porres, J.M.; Frías, J.; Vidal-Valverde, C.; Aranda, P. Nutritional assessment of raw and germinated pea (Pisum sativum L.) protein and carbohydrate by in vitro and in vivo techniques. *Nutrition* **2005**, *21*, 230-239, doi:10.1016/j.nut.2004.04.025.
26. Chinma, C.E.; Abu, J.O.; Adedeji, O.E.; Aburime, L.C.; Joseph, D.G.; Agunloye, G.F.; Adebo, J.A.; Oyeyinka, S.A.; Njobeh, P.B.; Adebo, O.A. Nutritional composition, bioactivity, starch characteristics, thermal and microstructural properties of germinated pigeon pea flour. *Food Bioscience* **2022**, *49*, 101900, doi:10.1016/j.fbio.2022.101900.
27. Joshi, S.; Bathla, S.; Singh, A.; Sharma, M.; Stephen Inbaraj, B.; Sridhar, K. Development of mung bean (Vigna radiate L.)-based next-generation vegan milk: Processing, nutritional composition and quality attributes. *International Journal of Food Science & Technology* **2023**, *58*, 785-794, doi:doi.org/10.1111/ijfs.16233.
28. Sattar, A.; Neelofar; Akhtar, M.A. Irradiation and germination effects on phytate, protein and amino acids of soybean. *Plant Foods for Human Nutrition* **1990**, *40*, 185-194, doi:10.1007/BF01104141.
